# Bedtime procrastination during the second COVID-19 lockdown in Portugal

**DOI:** 10.5935/1984-0063.20220031

**Published:** 2022

**Authors:** André Oliveira, Beatriz Pereira, Pedro Rosário, Paula Magalhães

**Affiliations:** Psychology Research Center, School of Psychology, University of Minho, Braga, Portugal.

**Keywords:** Bedtime Procrastination, Sleep Delay, COVID-19, Lockdown, Home Confinement

## Abstract

**Introduction:**

Bedtime procrastination is the deliberate delay of the time an individual goes to bed in the absence of external reasons. The COVID-19 pandemic has pushed families to establish new routines and ways of managing newfound roles and responsibilities. This scenario is likely to exacerbate bedtime procrastination due to, for example, a challenge in balancing professional and personal life.

**Objective:**

The aim is to present preliminary findings regarding bedtime procrastination and its relation to sociodemographic characteristics, sleep routines, perceived daily fatigue, dinnertime, and activities performed near bedtime, during the second lockdown in Portugal.

**Material and Methods:**

A cross-sectional online survey was conducted with a sample of 560 adults.

**Results:**

During home confinement, most people (79.46%) delayed their bedtime. However, this delay does not seem to be affecting the number of hours of sleep, as 88.60% were sleeping the recommended or appropriate number of hours. Nevertheless, most of the participants reported feeling tired throughout the day (53.04%), and individuals who reported to have procrastinated their sleep are those who reported more tiredness (*r*_pb_=.33, *p*<.01). Additionally, bedtime procrastination is positively associated with findings related to dinnertime (e.g., dinner between 9 p.m. and 10 p.m., *r*_pb_=.19, p<.01) and with engagement in activities near bedtime (e.g., studying/working, *r*_pb_=.39, *p*<.01).

**Conclusion:**

Current data shows relationships between bedtime procrastination and most of the studied variables. Specifically, findings indicate that lack of routines, especially scheduled nighttime routines (e.g., studying/working near bedtime), may have contributed to bedtime procrastination during the second lockdown in Portugal.

## INTRODUCTION

The current coronavirus disease 2019 (COVID-19) pandemic has obliged governments to implement measures likely to contribute to the balance between the protection and promotion of public health, the respect for the individuals’ rights, and the reduction of social and economic problems^1,2^. In Portugal, as in other countries, to prevent the waves of COVID-19 cases and protect the population, the government has implemented a series of national lockdown measures, such as most people having to engage in remote working^3^. Although these health-related measures and efforts are necessary to reduce the spread of infections and the pressure on the healthcare systems, they are also likely to exacerbate previously dormant or unnoticed personal issues and health conditions. One such example is the decrease in quality of sleep^4^. Recent reports have indicated that sleep disturbances and deprivation are escalating with the COVID-19 pandemic^5,6^. In fact, worldwide there has been an increase in sleep problems leading to higher consumption of sleeping pills, Portugal included^7^. These findings pose a serious health concern due to the critical role sleep plays in maintaining individuals’ homeostasis, health, and overall functioning and well-being^8^.

Previous studies suggest that about 40% of individuals report sleeping less than international guidelines recommendations^9^, despite 90% of them not suffering any kind of sleep disorder^10^. Causes for sleep insufficiency are manifold (e.g., psychological distress, behavioral), with one possibility being that individuals are procrastinating their bedtime and, consequently, sleeping fewer hours than recommended^11-13^. This highlights the need to investigate reasons for sleep insufficiency other than sleep disorders, such as procrastination^11^. Procrastination is the unnecessary delay in performing a given task despite the detrimental impact of that delay on the final outcome, and may be understood as a failure of self-regulatory skills^14^.

Bedtime procrastination is the deliberate delay of the time an individual goes to bed, in the absence of external reasons justifying this behavior^12^. Previous research has shown that individuals may purposely postpone their sleep because they feel they deserve time for themselves, belief that delaying sleep will help them eventually to effortlessly fall asleep or even because they lose track of time when absorbed in other activities^15^. As the COVID-19 pandemic continues to force individuals to establish new routines, the periods of home confinement may exacerbate or even trigger factors for sleep delay and bedtime procrastination. For example, for many individuals, home confinement implied working from home and distance learning, which brought along associated challenges in reconciling time designated for work with leisure and family time^16^. Consequently, individuals may feel that besides the time spent at home working and unable to relax, they deserve some time for themselves, which may translate in delaying their bedtime. Additionally, the pandemic has led to an increased use of digital media and other immersive activities near bedtime, which further contributes to a diminished sense of time and temporal disorientation^4,17^. Lastly, during home confinement, individuals may feel an excess of energy/lack of tiredness near bedtime due to the increased proximity of dinner to the time to go to bed, which leads to a sense of fullness that inhibits one’s ability to fall asleep^4,18,19^. Considering the variables that have emerged with the enforcement of COVID-19 home confinement, the main aim of the current study is to present preliminary findings regarding the bedtime procrastination phenomenon and its relationship with sociodemographic characteristics, sleep routines, perceived daily fatigue, time of dinner, and activities participated in near bedtime, as reported during the second lockdown in Portugal.

## MATERIAL AND METHODS

### Participants

According to the 2020 estimation of the Portuguese population conducted by the *Instituto Nacional de Estatística* (INE), 8,379,183 adults reside in Portugal, which is the universe of the present study. The sample size for this study was calculated with the online calculator *Netquest*®. Considering the population size indicated, with 50% heterogeneity, 98% reliability, and a 5% margin of error, 542 participants were estimated to be included in the study.

The survey was opened by 1,101 people and was fully completed by 685 subjects (completion rate of 62.2%), between January 25 and February 10, 2021. From this sample, 125 participants were excluded due to factors likely to have an impact on sleep insufficiency^15,20,21^ as follows: i) having a physical or psychological health problem that affects sleep (*n* = 78), ii) working in shifts (*n* = 24), iii) having a sleep disturbance (*n* = 10), and iv) taking care of a child under the age of three (*n* = 13) (see sociodemographic data and exclusion criteria section for more details about exclusion criteria assessment). The final sample consisted of 560 participants.

### Measures

#### Sociodemographic data and exclusion criteria

The socio-demographic questionnaire included questions about age, gender, educational status, and professional situation. Besides, to verify the exclusion criteria, participants were asked to complete a self-reported questionnaire regarding whether they: 1) “have a physical and/or psychological health problems that affect sleep diagnosed by a professional (e.g., restless legs syndrome, depression, obsessive-compulsive disorder)”; 2) “work in shifts”; 3) “have health problems related to sleep diagnosed by a professional (e.g., obstructive sleep apnea, sleepwalking)”; and 4) “are responsible for children under the age of three”^15,20,21^. These four questions were answered on a dichotomous scale (yes/no), and the participants who answered “yes” to at least one of these four questions were excluded from the analyses. These participants were excluded from the data analyses due to the only interest in accessing the relationships between bedtime procrastination and a set of variables (e.g., perception of tiredness) irrespective of other conditions that could affect sleep.

#### Sleep routines variables

Information about sleep routines was collected through open-ended questions: the times that participants intend to go to bed and the actual time they go to bed, the time that participants fall asleep, the time that participants intend to wake up, and the actual time that they wake up.

Sleep routines during home confinement were analyzed as follows: bedtime delay (delay between the intended time to go to bed and the actual bedtime; e.g., intended bedtime: 10 p.m., actual bedtime: 11 p.m., bedtime delay: 1 hour), wake up delay (delay between the intended time to wake-up and the actual wake-up time; e.g., intended wake-up time: 6 a.m., actual wake-up time: 8 a.m., wake-up delay: 2 hours), and sleep time duration (time between the actual fall asleep and wake up times). Following the National Sleep Foundation’s sleep time duration recommendations^22^, participants’ sleep hours were categorized according to their age groups (i.e., young adults, adults, older adults) as ‘recommended’, ‘may be appropriate’ or ‘not recommended’. To illustrate, for an young adult aged between 18 and 25 years, it is ‘recommended’ to sleep between 7 and 9 hours per night, it ‘maybe appropriate’ to sleep 6 hours or 10-11 hours, and it is ‘not recommended’ to sleep 5 hours or less or 12 hours or more per night (for other age groups’ recommendations see Hirshkowitz et al. (2015)^22^).

#### Perception of tiredness

To evaluate daytime perceived fatigue, an adapted version of the “daytime fatigue” from Kroese et al. (2016)^12^ was used. Participants were asked if they felt tired throughout the day, and they answered on a dichotomous scale (yes/no).

#### Dinnertime and activities carried near bedtime

Dinnertime was evaluated through an adapted version of the routine-related/contextual variables from the works of Magalhães et al. (2020, 2021)^13,23^. Participants were asked about the time interval that they usually have dinner (e.g., before 7 p.m., between 9 p.m. and 10 p.m., after 11 p.m.), of which they were asked to respond to each interval on dichotomous scale (yes/no). The engagement in activities before going to bed was assessed by asking participants, from a list of activities, to select those in which they usually engage in before bedtime. Based on the literature^13^ we singled out 7 activities (e.g., listening to music, playing games, eating). However, because the Magalhães et al. (2020)^13^ list of activities was built for a sample of Portuguese late adolescents/young adults, a group of researchers from distinct backgrounds (e.g., educational, clinical psychology, and medicine), and research seniority (e.g., full professor, junior researchers, PhD students) met to adjust this list to the target population of the current study (i.e., Portuguese adults). Finally, the team agreed on a final list with 14 activities (e.g., thinking/planning next day, meditating/praying, spending time with family, doing a beauty/hygiene ritual). Participants answered to each activity on a dichotomous scale (yes/no). Additionally, two options were presented to participants: “I don’t do anything, and I go to bed” and “other”. For this study, the type of activities as well as the number of activities in which the participants engaged in before going to bed were considered.

#### Bedtime procrastination

Bedtime procrastination was evaluated through the “Bedtime Procrastination Scale” developed by Kroese et al. (2014, 2016)^11,12^. For the present study, the original scale was adapted to Portuguese. A factor analysis was conducted and the Kaiser-Meyer-Olkin measure verified the adequacy of the sample for the analysis with the value of KMO = .93. Bartlett’s test of sphericity χ^2^ (36) = 2512.902 was significant (*p* < .001) and indicated that it was appropriate to apply a principal component analysis. Data showed a solution with one factor, accounting for 56.4% of the variance. The final questionnaire is a nine-item instrument (e.g., “I go to bed later than I had intended”) and items were answered in a five-point Likert-like scale from 1 [(almost) never] to 5 [(almost) always]. Total scores ranged between nine and 45, with higher scores indicating more frequent engagement in bedtime procrastination (α = .90).

### Procedure

Participants completed an anonymous cross-sectional online survey, through the Qualtrics XM^24^ survey platform, after reading the online consent form and explicitly agreeing to participate in the survey. The survey was shared via social media and e-mail, targeting Portugal’s residents over 18 years old. There was no monetary or credit compensation for participating in the study. The study protocol was approved by the University of Minho Ethics Committee for Research in Social and Human Sciences (CEICSH 087/2020) and was conducted in accordance with the Declaration of Helsinki. Data reported in this study are part of a wider research project designed with multiple purposes regarding the impact of home confinement on sleep procrastination.

### Data analysis

Data were treated and analyzed with IBM® SPSS® 27 version on Catalina®^25^. Descriptive statistics and frequency analyses were conducted. Factorial analysis including the KMO and Bartlett Test, the Communalities test, the total explained variance test, the component matrix, and the reliability test were conducted on the “Bedtime Procrastination Scale”^11,12^. For the purpose of data analysis, the nominal variables professional situation, sleep time duration categories, dinnertime interval, and activities carried out near bedtime were transformed into dichotomous nominal variables (e.g., working: 0 = no, 1 = yes; recommended sleep time duration: 0 = no, 1 = yes; dinner before 7 p.m.: 0 = no, 1 = yes; Doing nothing, and going to bed: 0 = no, 1 = yes). Pearson (*r*) and Point-Biserial (*r*_pb_) correlations were conducted between the bedtime procrastination and sociodemographic data, sleep routines variables, perception of tiredness, activities carried out near bedtime, and dinnertime intervals. Correlations’ effect sizes were analyzed according to Cohen’s suggestions^26,27^.

## RESULTS

### Sociodemographic data

Table 2 shows the correlations between sociodemographic data and bedtime procrastination. Data show positive and negative relationships, all with small effect sizes. A positive relationship between bedtime procrastination and being a student (*r*_pb_ = .26, *p* <.01) was found. Bedtime procrastination was negatively correlated with age (*r* = -.22, *p* <.01), being employed (*r*_pb_ = -.22, *p* <.01), and being responsible for a child over three years old (*r*_pb_ = -.11, *p* <.01).

**Table 2:** Correlation coefficients between bedtime procrastination and sociodemographic variables.

	r	Effect size
Age	-.22**	small
	**r_pb_**	**Effect size**
Gender	-.02	
Professional situation		
Working	-.22**	small
Unemployed	.01	
Studying	.26**	small
Retired	-.05	

**Note:**

** *p* < .01,

* *p* < .05, gender (0 = men, 1 = women), professional situation categories (0 = no, 1 = yes).

### Sleep routines variables

Results indicate that there is a delay in bedtime and wake-up time [*M*_bedtime delay_ = 1.41 hours (*SD* = 1.25); *M*_wake-up delay_ = .64 hours (*SD* = 1.60)]. Concerning the bedtime delay, 445 (79.46%) participants reported delaying their bedtime, 72 (12.86%) reported going to bed at the time that they intended to, and 10 (1.79%) reported anticipating bedtime in relation to the time that they intended to go to bed (see Table 3). On average, participants intended to go to bed at 10:58 p.m. (*M* = 22.97 hours, *SD* = .84) but reported to lay down at 0:23 a.m. (*M* = 24.37 hours, *SD* = 1.55).

**Table 3:** Frequency of participants reporting bedtime and wake-up delay.

	Anticipation 3 or more hours	Anticipation 2 hours	Anticipation 1 hour	Anticipation up to 1 hour	No anticipation or delay	Delay up to 1 hour	Delay 1 hour	Delay 2 hours	Delay 3 or more hours
Bedtime delay	-	1 (.18%)	6 (1.07%)	3 (.54%)	72 (12.86%)	88 (15.71%)	177 (31.61%)	110 (19.64%)	70 (12.50%)
Wake-up delay	5 (.89%)	21 (3.75%)	61 (10.89%)	42 (7.50%)	101 (18.04%)	94 (16.79%)	130 (23.21%)	61 (10.89%)	40 (7.14%)

Regarding the results of the wake-up delay, 325 (58.04%) participants reported delaying their wake-up time. What is more, 101 (18.04%) did not anticipate or delay waking-up, and 129 (23.04%) anticipated the time to wake-up (see Table 3). On average, participants intended to wake-up around 8:05 a.m. (*M* = 8.08 hours, *SD* = .94), but reported to wake-up around 8:43 a.m. (*M* = 8.72 hours, *SD* = 1.81). Matching data against the sleep time duration recommendations of the National Sleep Foundation’s^22^, 407 (72.70%) participants slept within the recommended, 89 (15.90%) slept for a period of time that may be appropriate, and 42 (7.50%) did not successfully achieve the number of hours of sleep recommended (see Table 4).

**Table 4:** Frequency of participants sleep time duration accordingly to the recommendations.

	Recommended	May be appropriate	Not recommended
Young adults (18-25 years old)	228 (40.70%)	52 (9.30%)	19 (3.40%)
Adults (26-64 years old)	176 (31.40%)	35 (6.30%)	23 (4.10%)
Older adults (≥65 years old)	3 (.50%)	2 (.50%)	-
General (all ages)	407 (72.70%)	89 (15.90%)	42 (7.50%)

Table 5 presents the correlations between sleep routine variables and bedtime procrastination. Positive correlations with large effect sizes were found between bedtime procrastination and bedtime delay (*r* = .70, *p* <.01), the actual bedtime (*r* = .65, *p* <.01), and the fall asleep time (*r* = .64, *p* < .01). In addition, there were significant positive correlations with medium effect sizes for bedtime procrastination with the actual wake-up time (*r* = .45, *p* <.01) and the wake-up delay (*r* = .40, *p* <.01). Data show positive correlations with small effect sizes between bedtime procrastination and the ‘not recommended’ sleep time duration (*r*_pb_ = .24, *p* <.01), the intended bedtime (*r* = .18, *p* <.01), and the intended wake-up time (*r* = .17, *p* < .01). Lastly, negative correlations with small effect sizes were found between bedtime procrastination and the ‘recommended’ sleep time (*r*_pb_ = -.22, *p* < .01) and the sleep time duration (*r* = -.18, *p* < .01).

**Table 5:** Correlations coefficients between bedtime procrastination and the sleep routines variables.

	r	Effect size
Intended bedtime	.18**	small
Actual bedtime	.65**	large
Fall asleep time	.64**	large
Intended wake-up time	.17**	small
Actual wake-up time	.45**	medium
Bedtime delay	.70**	large
Wake-up delay	.40**	medium
Sleep time duration	-.18**	small
	**r_pb_**	**Effect size**
Sleep time duration categories		
Recommended	-.22**	small
May be appropriate	.09*	
Not recommended	.24**	small

**Note:**

** p < .01,

* p < .05, sleep time duration categories (0 = no, 1 = yes).

### Perception of tiredness

Although most of the participants’ reported sleep hours that fell within the recommended time interval^22^, more than half of the sample (i.e., 53.04%) reported feeling tired throughout the day (see Table 6). In addition, there was a significant positive correlation with medium effect size between bedtime procrastination and feeling tired throughout the day (*r*_pb_ = .33, *p* < .01).

**Table 6:** Perception of tiredness frequency and its correlation coefficient with bedtime procrastination.

	n	%
Feel tired throughout the day		
Yes	297	53.04
No	260	46.43
	**r_pb_**	**Effect size**
Feel tired throughout the day	.33**	medium

**Note:**

** p < .01, ‘feel tired throughout the day’ for the correlation (0 = no, 1 = yes).

### Dinnertime and activities carried near bedtime

Regarding the number of activities that participants reported to get involved with near bedtime (*M* = 5.08 activities, *SD* = 2.42), more than half (*n* = 313, 55.83%) reported involvement in five or more different activities. Particularly, the most reported activities engaged in were that of ‘using social networks’ (*n* = 398, 71.07%), ‘watching movies/series on streaming platforms’ (*n* = 308, 55.00%), and ‘watching television’ (*n* = 275, 49.11%). Lastly, 39 (6.96%) participants reported not to engage in any activity around bedtime.

Table 7 shows the correlations between bedtime procrastination and dinnertime and the set of activities carried out near bedtime. Data show positive significant correlations with a medium effect size between bedtime procrastination and the number of activities participants were engaged in near bedtime (*r* = .30, *p* < .01) and studying/working near bedtime (r_*pb*_ = .39, p < .01). Significant positive correlations with small effect sizes were found between bedtime procrastination and ‘eating near bedtime’ (r_*pb*_ = .28, p < .01), ‘watching videos’ (r_*pb*_ = .27, p < .01), ‘using social networks’ (r_*pb*_ = .23, p < .01), ‘sending messages or doing calls/videocalls’ (r_*pb*_ = .20, p < .01), ‘watching movies/series on streaming platforms’ (r_*pb*_ = .20, p < .01), dining between 9 p.m. and 10 p.m. (r_*pb*_ = .192, p < .01), ‘listening to music’ (r_*pb*_ = .18, p < .01), ‘playing games’ (r_*pb*_ = .16, p < .01), dining between 10 p.m. and 11 p.m. (r_*pb*_ = .12, p < .01), and ‘thinking/planning the next day’ (r_*pb*_ = .11, p < .01). At last, significant negative correlations, with small effect sizes, were found between bedtime procrastination and ‘doing nothing and going to bed’ (r_*pb*_ = -.29, p < .01), dining between 7 p.m. and 8 p.m. (r_*pb*_ = -.17, p < .01), and ‘watching television’ (r_*pb*_ = -.15, p < .01).

**Table 7:** Correlations coefficients between bedtime procrastination and the following variables: dinner time and, activities carried near bedtime.

	r	Effect size
Number of activities carried near bedtime	.30**	medium
	**r_pb_**	**Effect size**
Dinner…		
…before 7 p.m.	-.00	
…between 7 p.m. and 8 p.m.	-.17**	small
…between 8 p.m. and 9 p.m.	-.03	
…between 9 p.m. and 10 p.m.	.19**	small
…between 10 p.m. and 11 p.m.	.12**	small
…after 11 p.m.	.07	
Doing nothing, and go to bed	-.29**	small
Using social networks	.23**	small
Reading	-.07	
Studying/working	.39**	medium
Watching videos	.27**	small
Watching movies/series on streaming platforms	.20**	small
Watching television	-.15**	small
Listening to music	.18**	small
Thinking/planning next day	.11**	small
Meditating/praying	-.08	
Spending time with family	-.06	
Sending messages or make calls/videocalls	.20**	small
Playing games	.16**	small
Eating	.28**	small
Talking to someone else in my room	-.03	
Doing a beauty/hygiene ritual	-.02	

Note:

** p < .01, dinner interval (0 = no, 1 = yes), activity carried near bedtime (0 = no, 1 = yes).

## DISCUSSION

This cross-sectional study investigated bedtime procrastination-related factors during the second lockdown in Portugal. Preliminary findings show that during home confinement, most people (i.e., 79.46%) delayed their bedtime. However, this delay does not seem to be affecting the total number of hours of sleep. In fact, 88.60% of the participants reported to have slept the recommended, or what may be deemed appropriate, number of hours of sleep according to the National Sleep Foundation’s recommendations^22^. This result is surprising in comparison to prior research performed pre-pandemic, of which data had indicated that individuals who procrastinate their bedtime tend to sleep less hours than the recommended, i.e., experience sleep insufficiency^11^. The present results may be explained in part by the concurrently experienced phenomenon during lockdown in which individuals are also waking up later than the intended hour. In fact, more than half of our sample reported to have been postponing their wake-up time by approximately 39 minutes. Thus, although bedtime procrastination does not appear to be influencing the number of hours of sleep during the lockdown, it is possible that bedtime procrastination is affecting individuals’ morning routines, and consequently, the various subsequent daily activities. One possible indicator of the impact of bedtime procrastination is that most of the participants reported feeling tired throughout the day, and along this line of questioning, the individuals who reported to procrastinate their sleep more are those who also reported more tiredness.

The present preliminary findings suggest that the lack of routines, especially nighttime routines, could be a major cause of bedtime procrastination. Current data indicate that younger individuals reported experiencing more extreme bedtime procrastination. This result aligns with previous research showing that general and bedtime procrastination habits slightly decrease with age^28^. In fact, with the advancement of age, people are likely to develop strategies necessary to cope with the responsibilities related to work schedules and family life, leaving less opportunities for procrastination^28^. Accordingly, the present results suggest that students are more prone to bedtime procrastination. In fact, pre COVID-19 data has already emphasized the notion that students report more bedtime procrastination than the general population^29^. However, we further hypothesize that the lack of regular class schedules and social interactions may be exacerbating students’ sleep procrastination under the lockdown restrictions. Regarding nighttime routines, we found a relationship between dinnertime and bedtime procrastination. Specifically, it seems that individuals having dinner between 9 p.m. and 11 p.m. are more likely to postpone going to bed. This data is consistent with previous research that indicates that eating later than usual, i.e., closer to bedtime, is more common during confinement, and could contribute to an increased time required to fall asleep^30-33^. Thus, it is possible that individuals are strategically delaying bedtime if they feel that the sensation of fullness would impede on their ability to fall asleep earlier. Additionally, bedtime procrastination is positively associated with engaging in activities near bedtime, especially studying and working. Our results suggest that individuals may be struggling with work/study-life balance, such as the ability to implement and enforce schedule limits. Literature shows that engaging in immersive activities, such as working or studying, contributes to a loss of perception of the passage of time, which could contribute further to bedtime procrastination^15^.

Considering the uncertainty associated with the duration and continued course of the COVID-19 pandemic, including the fourth wave and new lockdowns that Europe is facing, future research and interventions should acknowledge this phenomenon of bedtime procrastination. Bedtime procrastination can be understood as a failure in self-regulation processes^12^, and therefore promoting the practice of self-regulatory skills (e.g., time management) to achieve self-set goals (e.g., going to bed at an established hour) could be critical to curbing bedtime procrastination and its effects during the pandemic. In sum, despite promising, current findings merit further attention from researchers. Future research may further investigate what could explain bedtime procrastination during the lockdown, but also in daily lives with no restrictions.

### Limitations and future directions

Despite the promising results, this study has some limitations. First, the measurement of the variables with self-reported questionnaires is likely to lead to recall and social desirability biases. To minimize this concern, self-reported measures previously validated in studies with similar samples were used^12^. Future studies could consider combining self-reported measures with motion-based measures (e.g., actigraphy), that provides data with increased precision, quality, and completeness^34^. Moreover, the recruitment of participants took place via social media, and the data collection was online. Despite the several advantages of this method (e.g., possibility to collect data during a period of lockdown and social distancing, access to a large and diverse sample, less expensive and more efficient), there are some constraints that need to be acknowledged^35^. For example, the sample may not be entirely representative, as most social media users are young and well educated. Additionally, causal relationships between variables cannot be established due to the cross-sectional design of the study. Future research may consider a longitudinal design to infer causal relationship between variables. Lastly, future studies could consider assessing effects of bedtime procrastination on individuals’ daily routines and performances (e.g., implementing and maintaining family routines, work/study performance).

## Figures and Tables

**Table 1:** Participants’ characterization.

	n	Mean (SD)
Age	560	29.85 (12.83)
	**n**	**%**
Gender		
Men	142	25.4
Women	417	74.5
Other	1	.2
Age group		
Young adults (18-25 y)	311	55.5
Adults (26-64 y)	244	43.6
Older adults (≥ 65 y)	5	.9
Education level		
Basic education	2	.4
Secondary education	168	30
Undergraduate degree	167	29.8
Graduate degree	223	39.8
Professional situation*		
Working	235	42
Unemployed	25	4.5
Studying	321	57.3
Retired	6	1.1

**Note:**

* Non-mutually exclusive categories.
